# Cannabidiol use among elite-level Canadian athletes: the pursuit of improved sleep, pain relief, and enhanced recovery

**DOI:** 10.3389/fnut.2025.1711773

**Published:** 2025-12-05

**Authors:** Dimitri Karam, Erik Sesbreno, Kelly Drager, Susan Boegman, Lindsay R. Duncan, Dennis Jensen, Tyler A. Churchward-Venne

**Affiliations:** 1 Department of Kinesiology and Physical Education, McGill University, Montreal, QC, Canada; 2 Family Medicine Residency Program, Henry Ford Jackson Hospital, MI, United States; 3 Department of Nutrition, l’Institut National du Sport du Québec, Montreal, QC, Canada; 4 School of Human Nutrition, McGill University, Montreal, QC, Canada; 5 French-Speaking Olympic Sports Medicine Research Network, Paris, France; 6 Department of Nutrition, Canadian Sport Institute Alberta, Calgary, AB, Canada; 7 Department of Nutrition, Canadian Sport Institute Pacific, Victoria, BC, Canada; 8 Research Institute of the McGill University Health Centre, Montreal, QC, Canada; 9 Division of Geriatric Medicine, McGill University, Montreal, QC, Canada

**Keywords:** cannabidiol, nutraceutical, elite-level athletes, sleep, pain relief, recovery

## Abstract

**Introduction:**

Cannabidiol (CBD) is a compound in the cannabis plant with psycho-physiological effects that may support athletes’ training and recovery. Although not banned by the World Anti-Doping Agency, CBD products may carry a risk of inadvertent anti-doping violations due to contamination with prohibited cannabinoids. The *primary objective* of this study was to characterize the use, rationale, and perceived benefits of CBD use by elite-level athletes in Canada. The *secondary objectives* were to (1) identify the sources of information that influence CBD use, (2) describe how athletes are using CBD, and (3) explore the barriers or deterrents to its use.

**Design:**

Cross-sectional descriptive survey study.

**Methods:**

Elite-level Canadian athletes completed an anonymous online survey on CBD use between October 2021–June 2023.

**Results:**

80 athletes completed the survey. 38% (*n* = 30) had used CBD, with 30% (*n* = 9) of CBD users reporting active/current use. CBD users cumulatively agreed or strongly agreed that CBD is safe (96%); improved sleep (93%) and relaxation (90%); and reduced pain from training (77%). Friends (26%) and the internet (24%) were the most frequently reported first sources of information on CBD. Oral tincture/oil was the most commonly used (31%) form of CBD. The most reported reason for never using or discontinuing CBD was concern about an anti-doping rule violation (28%).

**Discussion:**

Given the self-reported benefits of CBD among elite-level Canadian athletes, alongside concerns about inadvertent anti-doping violations, clinicians working with this population should provide evidence-based guidance on CBD use and support informed decision-making to minimize risk and optimize athlete safety.

## 1 Introduction

Cannabidiol (CBD) is one among over 100 cannabinoids derived from the cannabis plant (1). CBD does not appear to produce cognitive impairment and appears generally safe and well tolerated in humans (2). A wide range of CBD-containing foods, dietary supplements, and cosmetic products are available online and over the counter around the world. CBD reportedly has anti-oxidative, anti-inflammatory, analgesic, and neuroprotective properties (3) that may have relevance for clinical and sport applications (4).

Although the World Anti-Doping Agency (WADA) removed CBD from its prohibited list in 2018 (5), it remains unclear if CBD products prescribed or marketed to athletes are sufficiently screened for the presence of other cannabinoids that are prohibited by WADA, specifically tetrahydrocannabinol (THC) (5). The use of CBD-containing foods, dietary supplements and/or cosmetic products may therefore have the potential to cause a positive drug test for athletes under anti-doping regulations. Nevertheless, self-reported rates of cannabis use and misuse among US collegiate and professional athletes are on the rise (6). Furthermore, a survey study on CBD use from the United Kingdom in 517 male professional rugby players reported a prevalence of 26% for self-directed aims such as pain management, anxiety and stress reduction, and improved sleep (7). However, information on CBD use by elite-level Canadian athletes from Olympic and Paralympic sport programs is currently unavailable.

The primary objective of this study was to characterize the use, rationale, and perceived benefits of CBD use by elite-level athletes in Canada. The secondary objectives were to (1) identify the sources of information that influence CBD use, (2) describe how athletes are using CBD, and (3) explore the barriers or deterrents to its use.

## 2 Methods

### 2.1 Study design

A private, anonymous, cross-sectional survey study design was used in accordance with the Checklist for Reporting Results of Internet E-surveys (CHERRIES) (8), and Proper Reporting of Evidence in Sport and Exercise Nutrition Trials (9). Athletes were recruited nationwide (i.e., within Canada) through the Canadian Olympic and Paralympic Sport Institutes Network. If approached directly by a member of the integrated support team, Registered Dieticians were the point of contact with athletes, thus providing them with the links to the online survey. If approached indirectly, a group e-mail notification, containing the links to the online survey, was sent by the communications lead of each institute. The survey was disseminated to over 1,000 nationally carded athletes, some of whom had Olympic experience, from different sports, including team sports, individual sports, artistic sports, and combat sports. Recruitment efforts took place during the in-season and off-season for various sports programs. To meet the inclusion criteria, athletes were required to be ≥ 18 years of age and compete as part of the 2021–2022 or 2022–2023 senior national (Olympic or Paralympic) Canadian team program. Additionally, athletes identified by the National Sport Organizations as part of the “NextGen” (Next Generation) program with the potential of qualifying for a roster position on the senior national team were also invited to participate. Targeted athletes would classify between tier 4 and 5 elite statuses based on available descriptive guidelines (10). Data were collected from October 15, 2021 to June 26, 2023. Assumed consent was provided upon electronic signing of the Information & Consent Form and submission of the survey. The study was approved by McGill University’s Faculty of Medicine and Health Sciences Institutional Review Board (IRB) (A09-B50-21B, 07/09/2021).

### 2.2 Survey design

The research team, comprising multiple disciplines, designed a self-administered and anonymous online/electronic survey hosted on the LimeSurvey (Version 3.28.52) platform. The content and face validity of the survey were assessed by both the team and experts versed in content and survey methodology. The usability and technical functionality of the electronic survey was tested before being released and available to the athletes to complete. To cater to the Canadian athlete community, the survey was made available in both official languages - English and French. The lead researcher (D. K.), carried out both the translation of the survey instrument into French and its subsequent back-translation into English. The survey was divided into 5 sections: (1) athlete information; (2) knowledge about CBD; (3) CBD consumption; (4) assumptions about CBD; and (5) further comments. Respondents were able to review and change their answers using a ‘previous’ button. No incentives were offered (e.g., monetary, prizes, or non-monetary incentives) for completing the survey. Surveys were submitted with a time-stamp to measure the time people needed to complete the survey; however, no time cut-off point was applied. A copy of the survey in both English and French can be found in Supplementary Tables 1, 2.

### 2.3 Data analysis

Only fully completed survey responses were included in the data analysis; survey responses which were terminated early were not analyzed. Missing data checks were performed to verify data integrity. Descriptive statistics (counts and percentages for categorical data) are presented for responses to closed-ended questions. We devised four major categories to classify sports activities, assigning participants to the category that best reflected the characteristics of their training and competition: endurance sports (Endurance); sports requiring primarily sprint power or skill (Sprint/Skill); team sports (Team); and sports with a focus on weight control (Weight) (11).

Data from multiple-choice questions allowing a single response are presented in figures (showing the number of responses) and/or described in the text using a combination of counts and proportions (%). Some figures were specifically designed to highlight trends by combining both percentage and count data for each answer choice. Percentages are rounded to the nearest whole number; in some instances, totals may not sum to 100%. For multiple-choice questions allowing multiple responses, data were detailed in the text similarly to single-response questions and/or displayed in figures. These figures present the proportion (%) of total selections attributed to each answer option for the question in focus. Data from type-in response questions were grouped into coherent categories and presented in figures and/or described in the text. A flowchart depicting participant progression through the survey study is shown in Supplementary Figure 1.

## 3 Results

**Figure 1:**
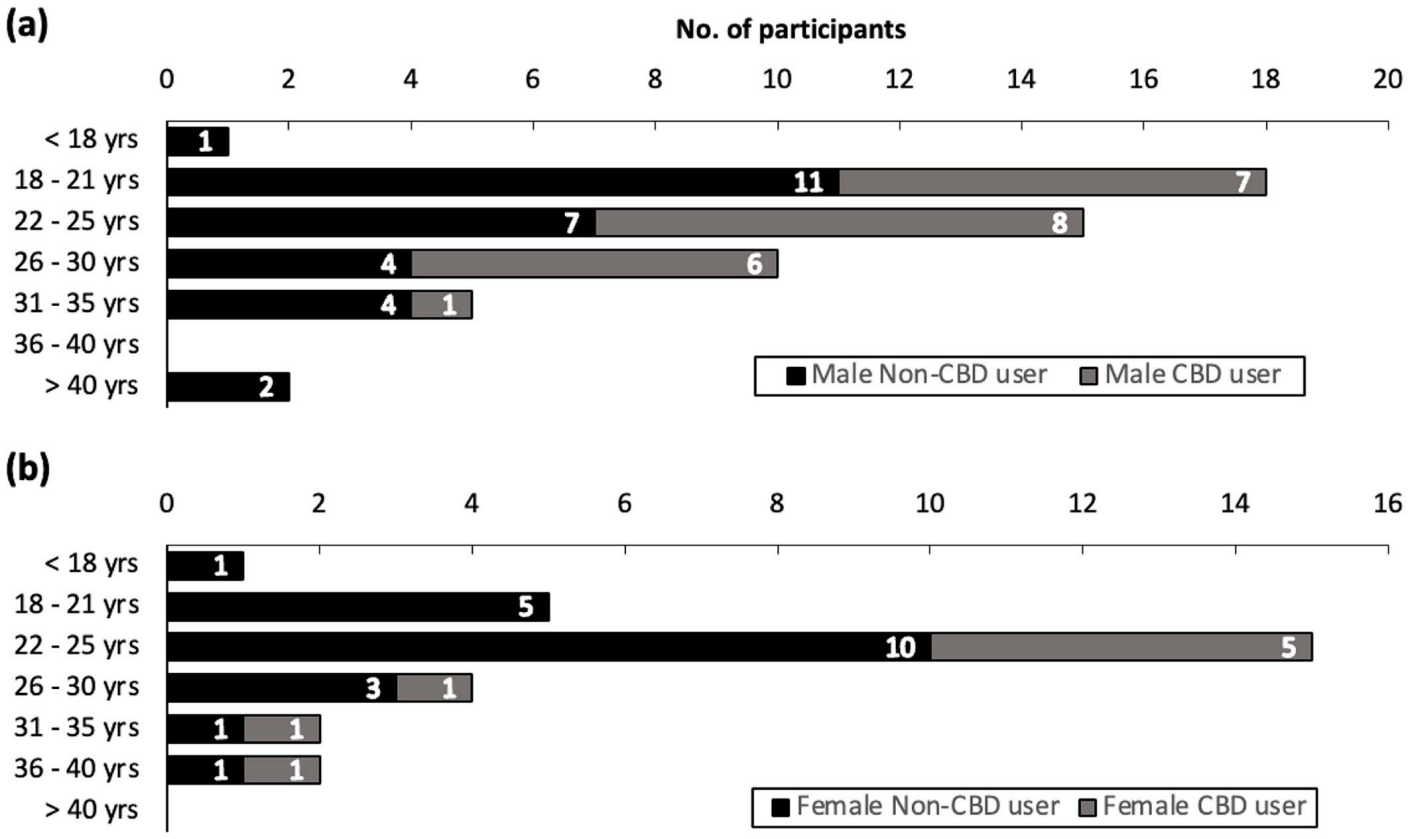
Distribution of participants by age group and CBD use status, stratified by consistent reporting of sex and gender identity for **(a)** males (*n* = 51) and **(b)** females (*n* = 29). CBD, cannabidiol.

**Figure 2:**
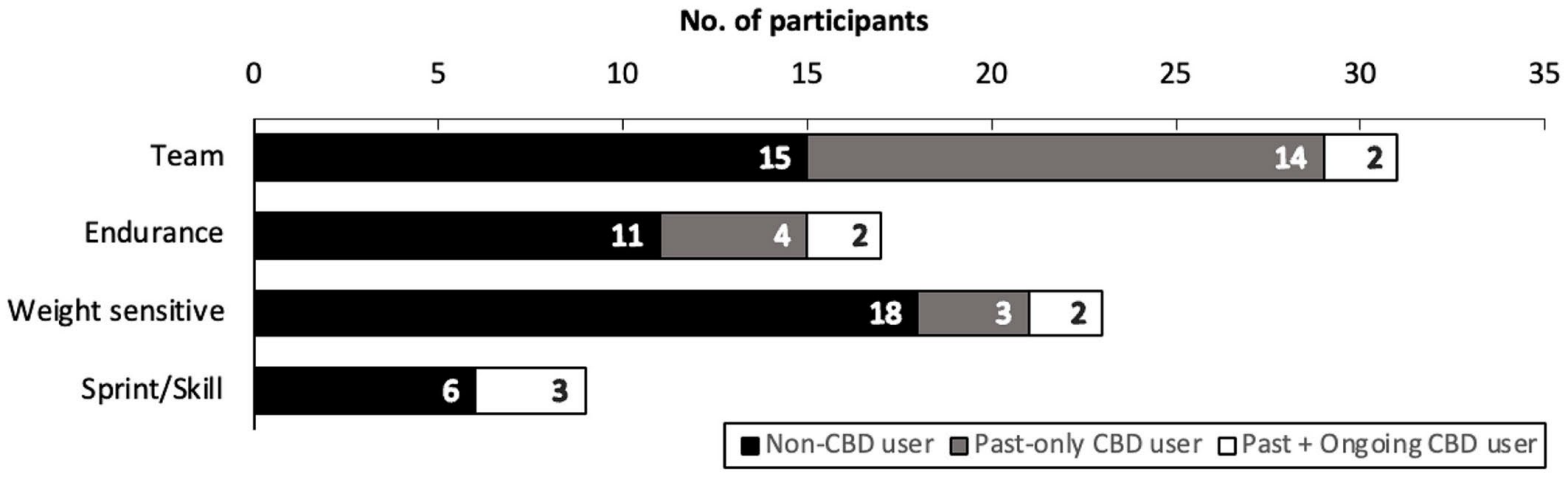
Number of participants and their CBD use status grouped by the type of sport that best describes their training and primary area of competition. CBD, cannabidiol.

One hundred and fifteen athletes accessed the online survey after signing the online consent form. Eighty elite-level athletes from 27 different national sporting organizations completed the survey (ratio of users who finished the survey/users who agreed to participate = 70%). Two athletes who completed the survey indicated meeting the eligibility of ≥ 18 years of age by signing the Information and Consent Form, but indicated that they were 16 and 17 years of age, respectively. Their data was retained following approval from the local IRB and is included in the overall study results. The 35 athletes who did not complete the survey discontinued the survey at various stages, yet most frequently at the start. Forty-four (55%) of the completed surveys were filled out in English, and the rest in French. Due to skip logic (i.e., adaptive questioning) implementation, the mean online survey time was 8 min. 45 s., with a median time of 6 min. 35 s., and a range from 2 min. 58 s. to 42 min. 20 s. Among athletes who completed the survey, 38% (*n* = 30) self-described as being CBD users, with 30% (*n* = 9) of users reporting active/current use. The proportion of CBD users between sexes, after stratifying for age, is shown in Figure 1a (male) and Figure 1b (female). The proportion of CBD users across groups of sports is shown in Figure 2. Among CBD users, 96% (*n* = 29) cumulatively agreed or strongly agreed that CBD was safe. Eight (10%) survey respondents were athletes from a paralympic sport program, of which 3 para-athletes self-described as being CBD users.

**Figure 3:**
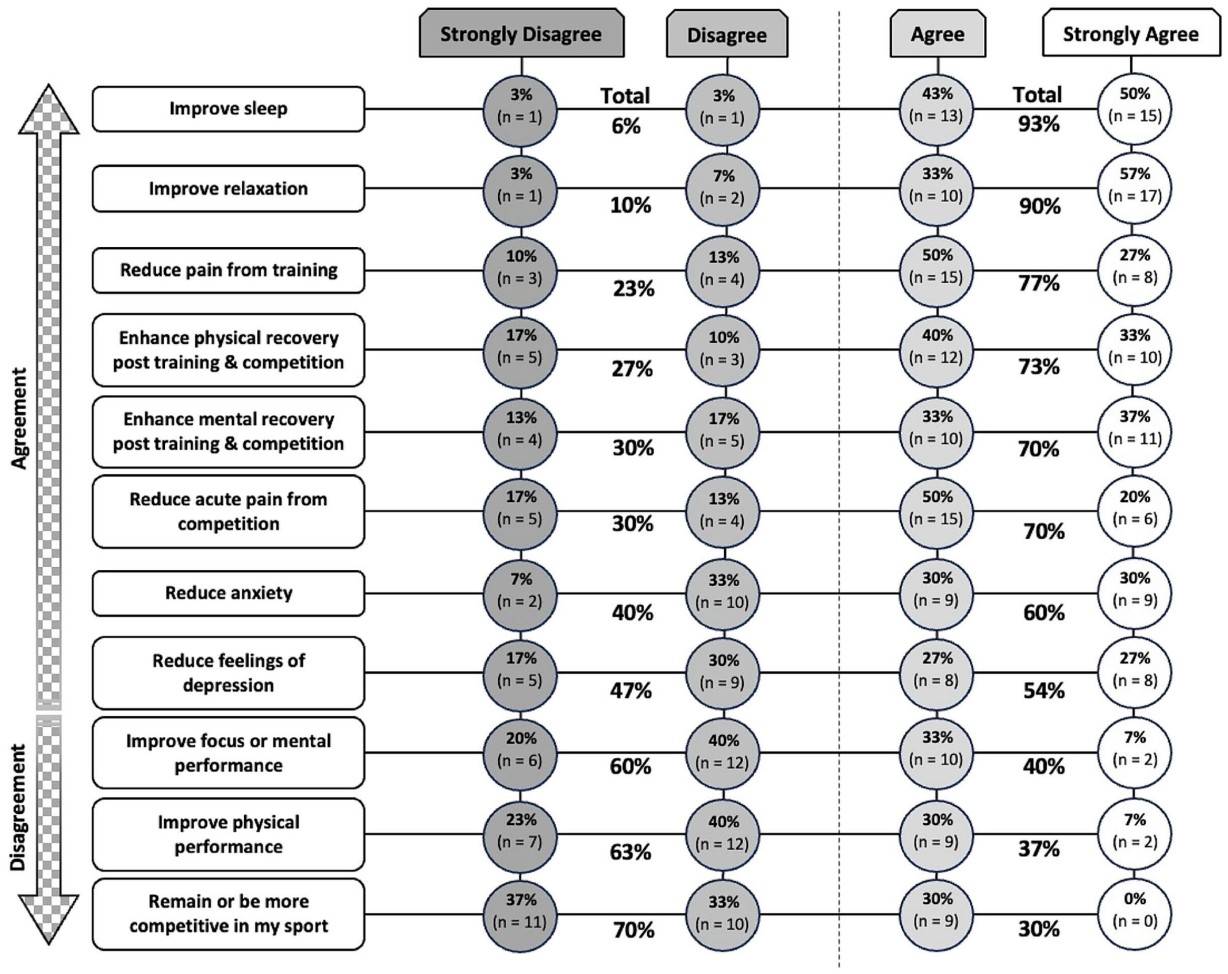
Ranking of intended CBD use scenarios among users (*n* = 30) from least to most agreed and disagreed upon. CBD, cannabidiol.

Intended CBD use scenarios among users (*n* = 30) are ranked in Figure 3, from least to most agreed and disagreed upon. CBD users cumulatively agreed or strongly agreed that CBD supplementation improved sleep (93%), improved relaxation (90%), and reduced pain from training (77%). CBD users also cumulatively disagreed or strongly disagreed that CBD supplementation improved focus or mental performance (60%), improved physical performance (63%), or helped them remain or be more competitive in their respective sport (70%).

**Figure 4:**
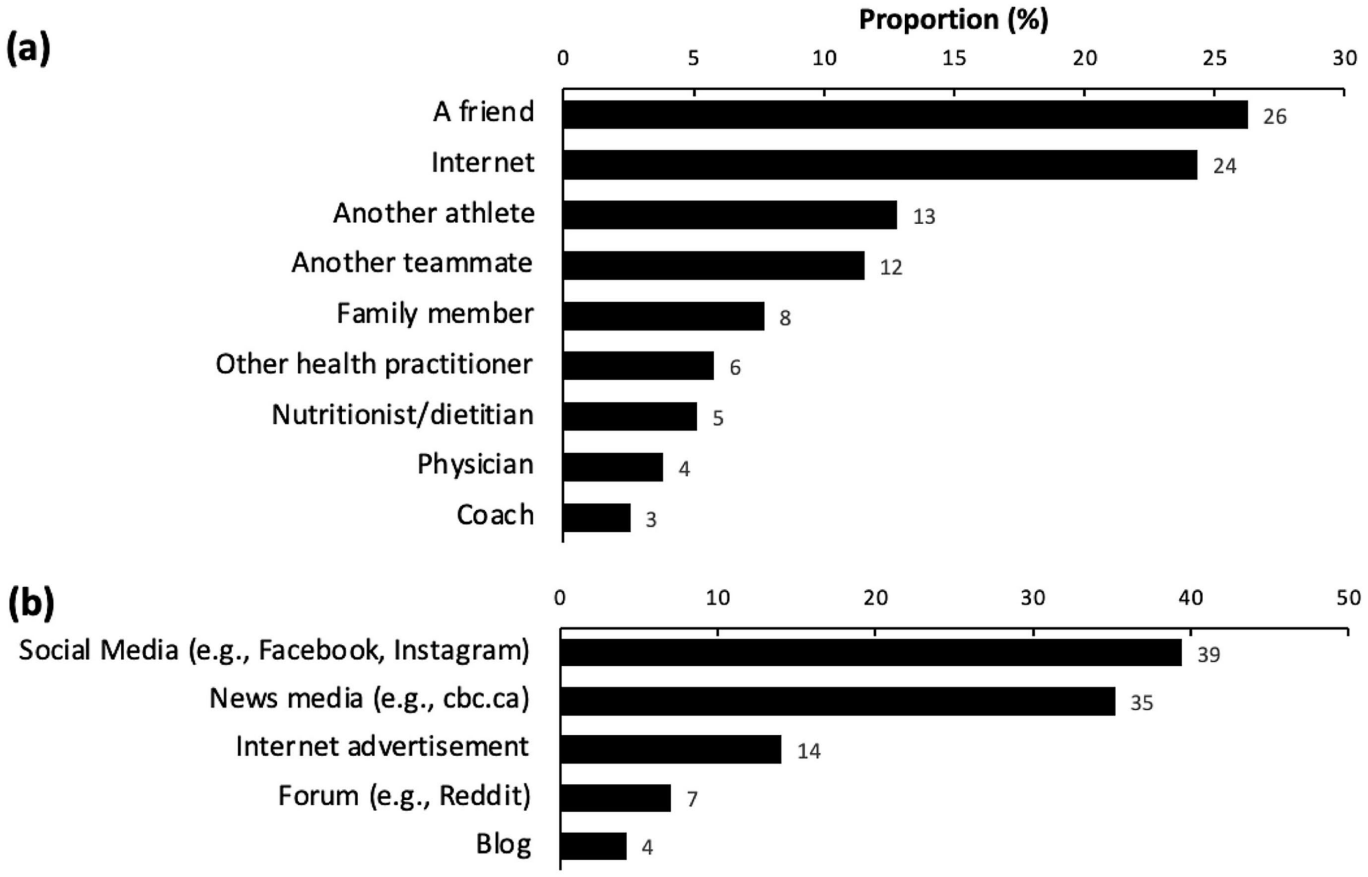
Orderly proportions of **(a)** sources introducing participants to CBD outside this survey (*n* = 71), and **(b)** specific breakdown of internet sources for athletes introduced to CBD online (*n* = 38). CBD, cannabidiol.

The different sources of information that first introduced participants to CBD are shown in Figures 4a,b. Among sources responsible for introducing CBD to athletes surveyed, a friend (26%) and the internet (24%) were the highest proportions. Among internet sources, social media (39%) and news media (35%) were the highest proportions. Among CBD users, the different forms of CBD used, the methods to determine an effective CBD dose, and the certainty of optimal CBD dosing are listed in Supplementary Figures 2a–c. The most reported method of CBD consumption was via a tincture or oil (31%), followed by inhalation (19%) (Supplementary Figure 2a). The most commonly reported method for determining an effective CBD dose was trial and error (55%) (Supplementary Figure 2b). Certainty in achieving optimal CBD dosing was at a median of 35%, with the majority (37%) of CBD users reporting 0% certainty (Supplementary Figure 2c).

The timing of CBD use in relation to training and competition cycles is shown in Supplementary Figures 3a,b. Thirty nine percent of CBD users consumed CBD after training, 55% in the evening prior to going to bed, and 5% before training (Supplementary Figure 3a). Fifty-two percent of CBD users consumed CBD during the off-season, 43% during the in-season outside of competition, and 5% during competition (Supplementary Figure 3b). Within the CBD user group, 73% (*n* = 22) could not recall the product brand used, 63% (*n* = 19) of athletes consumed <50 mg of CBD per dose, 7% (*n* = 2) of athletes consumed >100 mg CBD per dose, and 27% (*n* = 8) of athletes were unsure of the dose of CBD they consumed. Athlete-reported reasons for never using or for discontinuing CBD are listed in Supplementary Figure 4. The most reported reason for never using or for discontinuing CBD use was concern about an anti-doping rule violation (28%).

## 4 Discussion

The main findings of this study are: (1) 38% of athletes reported CBD use as a supplement, with 30% of users reporting active/current use; (2) athletes who used CBD cumulatively agreed or strongly agreed that the supplement improved sleep and relaxation, reduced pain from training, and enhanced physical and mental recovery after training or competition; (3) friends and the internet were major sources of information on CBD; (4) most athletes who used CBD were unable to recall the brand of CBD supplement they used, with the majority having used <50 mg of CBD per dose, and opted most commonly for the oral tincture/oil formulation and route of administration; and (5) the primary reason for discontinuing CBD use was fear of an inadvertent anti-doping rule violation due to CBD product contamination with THC and/or other banned cannabinoids.

### 4.1 Use of CBD

This is the first investigation on CBD supplement use among elite-level Canadian athletes. There was a relatively higher proportion of CBD users (38%) in this sample compared to a group of 517 professional male rugby players (26%) in the United Kingdom (7). It was recently reported that liberal attitudes and higher knowledge of cannabinoid use was associated with CBD use in a group of self-described multi-national elite-level endurance athletes (12). Although CBD users in this sample were observed across all sport types, a higher percentage of users to non-users was observed among team-based athletes. It is unclear if this was attributed to differences in athletes’ attitudes and knowledge of CBD use, sampling bias, and/or a response for peer-acceptance and cohesiveness that associate with attitudes toward risky behaviors such as marijuana use in team-based athletes (13). Sport science and medical personnel working in elite sports need to screen for athlete interest in using CBD to assess and possibly increase athletes’ knowledge on the topic.

### 4.2 Expected and perceived benefits of CBD use

There is a paucity of information on the expected and perceived benefits of CBD supplementation by elite-level athletes. More than 87% of individuals (*n* = 111) who habitually use cannabis and regularly participate in aerobic and/or resistance exercises perceive that cannabis, CBD, or THC assist them with recovery from exercise (14). In a previous study, 61% (*n* = 301) of adult community-based athletes indicated that they use cannabis for pain (15). For example, daily application of 20 mg CBD topical cream by retired professional American athletes (*n* = 20) from various sport disciplines was associated with a self-reported reduction in chronic pain from acute lower extremity injuries in a retrospective investigation (16). However, in a 2 × 2 factorial, randomized, placebo-controlled crossover trial, the analgesic effect of CBD was reported to be driven by both the pharmacological action of CBD and psychological expectancies of receiving CBD. Specifically, pain unpleasantness was reduced whenever participants received CBD, expected CBD, or both (17). Professional male rugby players reportedly use, or have used, CBD for the management of pain and recovery, sleep, anxiety, and other medical problems (i.e., concussion, chronic injuries, etc.) (7). A clinical observational trial reported that, in patients who consumed between 40–300 mg per day of CBD oil, there was an association with reduced non-cancer pain symptoms, lower anxiety and depression, and, in some users (12% of 253 patients), improved sleep (2). Earlier research also found that healthy male participants who ingested 400 mg of CBD reported lower subjective anxiety and increased mental sedation (18). Similarly, CBD users in the present study overwhelmingly agreed or strongly agreed that CBD improved sleep and relaxation, reduced pain from training, and enhanced both physical and mental recovery after training or competition. However, several studies to date have not found a beneficial effect of CBD use on muscle soreness and/or recovery of performance after exercise (19–22). For example, in untrained male participants consuming 150 mg of CBD oil per day, induction of muscle damage to the elbow flexors through maximal eccentric exercise did not result in any significant improvements in non-invasive markers of muscle damage, including perceived muscle soreness, arm circumference, and peak limb torque, across the three-day post-exercise recovery period (19). In the present study, many elite-level athletes reported using CBD dosages lower than those examined in controlled laboratory settings, and many were unsure of an optimal dosing protocol. Given the reported CBD dosages (*n* = 19, ≤50 mg; *n* = 2, >100 mg; *n* = 8 unsure; data not shown), it is plausible that CBD did not mediate the perceived reduction in pain from training, or the perceived enhanced physical recovery post-training or competition as reported by athletes. Alternatively, use of broad- or full-spectrum CBD products that also contain minor cannabinoids and terpenes (and up to 0.3% THC for full-spectrum CBD products), has been alleged to display increased effectiveness compared to isolated CBD via potential entourage effects (23). Therefore, the perceived benefits of CBD reported by athletes in our study may not only relate to the effects of CBD, but potentially also other compounds that may be present in CBD products acting additively or synergistically via entourage effects. Finally, reported effects could also be attributable to placebo effects or treatment expectation (24). Future dose–response studies examining the relationship between plasma CBD concentrations and sport-specific outcomes (e.g., sleep quality, muscle pain and soreness, and post-exercise recovery) are needed to better elucidate the effects of CBD in athletic populations. Additionally, further investigation into the cellular and molecular mechanisms underlying these effects is warranted to substantiate subjective reports from athletes regarding the perceived benefits of CBD use.

### 4.3 CBD and exercise performance

To date, CBD use has not been associated with direct exercise performance improvements under laboratory-based conditions (25–28). A semi-randomized crossover study found that inhaling aerosol from CBD-predominant cannabis within 20 min before exercise did not improve performance in an all-out 20-min cycling time trial among habitual cannabis users (25). In a randomized, double-blind, placebo-controlled crossover trial, physically active students who ingested 150 mg of CBD 90 min before a self-paced 10 km run showed no change in affective valence, as measured by the Feelings Scale (26). Additionally, in a randomized, double-blind, placebo-controlled trial, trained male endurance athletes who consumed 300 mg of CBD showed no improvement in time to exhaustion during an incremental treadmill run at a fixed speed (27). A randomized cross-over trial also reported that consuming 50 or 300 mg of CBD 1.5 h before a 60 min submaximal treadmill run followed by an incremental run to volitational exhaustion did not alter subjective experience of intensity of exercise or impact endurance performance (28). While short-term use of CBD has demonstrated equivocal effects on performance, somewhat aligning with our participants’ agreement that CBD may not improve physical performance, there is a need to investigate whether long-term CBD use influences physiological adaptations to different types of exercise (e.g., endurance- and resistance-type training). Additional rigorous intervention trials applying different training protocols and CBD dosing strategies, will help clarify the role of CBD on exercise performance and adaptations to training.

### 4.4 Sources of CBD information and the risk of inadvertent doping

Elite athletes in the present study reported using a range of sources to acquire information on CBD use. Participants more frequently relied on friends and internet sources (including social and news media) than on health professionals for information about CBD use. Young American adults aged 18 to 34 years also tended to rely more on friends and internet sources (e.g., social media) for information on CBD use (29). This is concerning, as Wysota and colleagues found that their sample was similarly misinformed about CBD, with users perceiving it more favorably for treating health issues (29). Social media, news outlets, and advertisement are unlikely to consistently provide accurate guidance on CBD use, given the frequent presence of illicit advertising and misinformation that mislead consumers about the risks and benefits of CBD use (30). Another important consideration for elite athletes is the influence of support personnel, including medical staff and coaches, on attitudes toward cannabinoid use. The perceptions of these professionals regarding CBD use may shape athletes’ willingness to discuss or disclose their own use and could influence both education and harm-reduction strategies. It has been reported that sports medicine physicians generally have a favorable attitude toward CBD and cannabis, but these perceptions are influenced by age, practice type and sex (31). Future research should examine how medical and coaching staff navigate the evolving landscape of CBD use in sport, particularly as social and regulatory norms continue to shift. Given that athletes frequently reported obtaining information on CBD from friends and online sources, there is also a need to test interventions that enhance evidence-based knowledge among both athletes and clinicians. For example, studies could trial educational materials or counseling sessions outlining the potential risks versus benefits of CBD, followed by assessments of changes in attitudes and behaviors. Such work would inform practical recommendations for supporting informed and responsible decision making on CBD use within athletic settings.

In addition to sources of information influencing CBD use, attention must also be directed toward its health implications within the context of sport and exercise, and risk of inadvertent doping. A recent review concluded that consuming CBD-containing foods and supplements may not offer substantiated health benefits and could pose potential health risks to consumers (32). Although no hepatotoxic effects were observed from a daily 60 mg oral dose of CBD during 7 days of intensive training, the relatively small dose did inhibit exercise-induced increases in glutamate oxaloacetate transaminase (GOT), also known as aspartate aminotransferase (AST), and glutamate pyruvate transaminase (GPT), also known as alanine aminotransferase (ALT), markers of liver activity (33). It is unclear if this could lead to adverse effects in CBD sensitive individuals. The use of commercially available CBD products, including those formulated with hemp oil, may result in the presence of cannabinoids or their metabolites in an athlete’s urine sample, potentially leading to an anti-doping rule violation (34, 35). A recent investigation revealed that daily use of a broad-spectrum CBD supplement resulted in detectable urine concentrations of WADA-prohibited cannabinoids with exercise appearing to increase concentrations of these cannabinoids (36). Unfortunately, most of the CBD users in the present study (73%) could not recall the brand of the CBD they used, while others reported using brands that could not be identified by the study authors (ES) in an online search to confirm 3rd party testing under the Informed Sport and NSF Sport website, as recommended in Canada’s best practice guidelines on dietary supplement use in sports (37). Moreover, even when verified as THC-free through testing, some CBD-fortified foods and beverages have been reported to convert CBD into THC under low pH conditions (pH < 4) at room temperature, potentially yielding sufficient THC (3 mg) to trigger a positive urine test for prohibited cannabinoids (38). Future studies should analyze CBD products athletes report using to test for THC and other cannabinoid concentrations and evaluate label accuracy.

Overall, the use of CBD in sport exists within a “grey zone” between permitted and prohibited cannabinoid use. Although CBD itself is not banned by WADA, many CBD products may contain trace amounts of THC and other cannabinoids, posing a risk of inadvertent anti-doping rule violations. This creates a fine line between unintentional doping and intentional use of cannabis products. Recent relaxations in WADA’s THC threshold further complicate this issue, as athletes may perceive cannabis use as less risky or even justify its use for perceived therapeutic benefits. It has been argued that using cannabis and cannabinoids does not meet the threshold for doping as there is a paucity of evidence for ergogenic effects, health risk, or violation of the spirit of sports (39). A clearer understanding of this “grey area” is essential to support athlete education on the risk of commercially available CBD products to avoid an inadvertent anti-doping rule violation, and ensure informed decision-making around cannabinoid use (40).

### 4.5 Limitations

Among the available pool of elite-level athletes for recruitment in Canada, we received a relatively small number of complete survey responses (*n* = 80). However, responses were collected from a diverse pool of elite-level Canadian athletes. It is possible that some athletes chose not to participate due to the lingering stigma surrounding cannabinoid use, despite CBD being permitted for use both in and out of competition. Those who agreed to participate may have had a greater interest or experience with CBD, which could introduce some selection bias. At the same time, this engagement may be viewed as a strength, as it ensured that data were gathered from athletes with meaningful insight into CBD use and attitudes within elite sport contexts. The anonymous design of the study limited the ability to thoroughly explore individual participants’ rationale for CBD use, perceived benefits, and decisions to consume it despite the risk of an anti-doping rule violation.

## 5 Conclusion

This study is the first to report on CBD use among elite-level Canadian athletes across multiple sport disciplines. Thirty eight percent of athletes self-reported using CBD, and cumulatively agreed or strongly agreed that CBD improved sleep and relaxation, reduced pain from training, and enhanced physical and mental recovery following training or competition. Friends and the internet were major sources of information on CBD and many athletes expressed concerns about the potential for inadvertent anti-doping rule violations due to CBD product contamination with THC or other banned cannabinoids. The insights provided by this survey may help guide future research into the effects of CBD use in athletic populations and the potential risks associated with CBD-containing products marketed to and used by athletes.

## Data Availability

The raw data supporting the conclusions of this article will be made available by the authors, without undue reservation.
