# Supplementary material for: Cannabidiol use among elite-level Canadian athletes: the pursuit of improved sleep, pain relief, and enhanced recovery
Source: Front Nutr. 2025 Dec 5;12:1711773. doi: 10.3389/fnut.2025.1711773 (PMC12715612; doi:10.3389/fnut.2025.1711773)
Supplement: Supplementary file 1 [file Table_1.docx]

**McGill Cannabidiol (CBD) Questionnaire**

**Probing The Use of Cannabidiol (CBD) by Elite-Level Athletes in Canada**

52 Questions Survey

**Section A. Athlete information**

**1. What is your age? ***

Please write your answer here:

|  |
| --- |

**2. What is your sex assigned at birth? ***
**(e.g. male, female, prefer not to disclose etc.)**
Please write your answer here:

|  |
| --- |

**3. What is your current gender identity? *
(e.g. man, woman, transgender, other)**

Please write your answer here:

|  |
| --- |

**4. What level do you compete at? ***

Please choose **all** that apply:

〇 Provincial

〇 National
〇 International level/Olympic

**5. Do you compete in USport? ***

Please choose **only one** of the following:

〇 Yes

〇 No

〇 Do not know

**6. Are you receiving sport science support and/or medical support through either a Canadian Sport Institute, l’Institut National du Sport du Québec or a Canadian Sport Centre? ***

Please choose **only one** of the following:

〇 Yes

〇 No

**7. How are you receiving funding for your sport? ***

Please choose **all** that apply:
〇 I am supporting myself

〇 I am a government sponsored athlete

〇 I am a corporate sponsored individual athlete

〇 I am a part of a corporate sponsored team

〇 I am a professional athlete

**8. Are you a parasport athlete? ***

Please choose **only one** of the following:

〇 Yes

〇 No

**9. What is the primary sport you compete in? ***

Please write your answer here:

|  |
| --- |

**10. Do you compete in any other sports? ***

**(****Based on your answer choice, certain subsequent questions may or may not be presented)**

***[Skip logic:*** *Show question 11 if the athlete answers “Yes” on question 10****]***

Please choose **only one** of the following:
〇 Yes

〇 No

**11. What other sports do you compete in?
(If you compete in multiple sports, separate the sports with a comma)**

***[Skip logic:*** *See question 10****]***
Please write your answer here:

|  |
| --- |

**12. How many people do you usually train with? ***Please choose **only one** of the following:
〇 None (I train alone)

〇 1

〇 2

〇 3

〇 4

〇 5

〇 6

〇 7

〇 8

〇 9

〇 10 or more

**Section B. Knowledge about CBD**

**13. Have you ever heard of** **Cannabidiol (CBD) prior to filling out this survey? ***

**(Based on your answer choice, certain subsequent questions may or may not be presented)**

***[Skip logic:*** *Show question 14 if the athlete answers “Yes” on question 13****]***

Please choose **only one** of the following:

〇 Yes

〇 No

**14. How did you first hear of Cannabidiol (CBD)? *
(Based on your answer choice, certain subsequent questions may or may not be presented)**

***[Skip logic:*** *See question 13****]***

***[Skip logic:*** *Show question 15 if the athlete answers “Internet” on question 14****]***

Please choose **all** that apply:

〇 Internet (e.g., social media, news media, or advertisement)

〇 Nutritionist/dietitian

〇 Physician

〇 Other practitioner (e.g., massage therapist, physiotherapist, chiropractor)

〇 A friend

〇 Another teammate

〇 Another athlete

〇 Family member

〇 Coach

〇 Other (please specify) _________________

**15. If you heard about Cannabidiol (CBD) on the internet, which source did you hear about it from?**

***[Skip logic:*** *See question 14****]***

Please choose **all** that apply:
〇 News media (e.g., cbc.ca)

〇 Blog

〇 Forum (e.g., Reddit)

〇 Social media (e.g., Facebook, Instagram)

〇 Internet advertisement

**Section C. CBD consumption**

**16. Have you ever used Cannabidiol (CBD)? ***

**(Based on your answer choice, certain subsequent questions may or may not be presented)**

***[Skip logic:*** *Show questions 17 to 42 if the athlete answers “Yes” on question 16****]***

Please choose **only one** of the following:

〇 Yes

〇 No

**17. Are you currently using Cannabidiol (CBD)? ***

***[Skip logic:*** *See question 16****]***

Please choose **only one** of the following:

〇 Yes

〇 No

**18.** **How long have/did you use(d) Cannabidiol (CBD) for? ***

**(e.g., 1 year and 6 months)**

***[Skip logic:*** *See question 16****]***

Please write your answer here:

|  |
| --- |

**19. If you used Cannabidiol (CBD) in the past, and either stopped completely or stopped for a period of time, what was the reason(s) for you discontinuing use? ***

***[Skip logic:*** *See question 16****]***

Please choose **all** that apply:

〇 Too expensive

〇 Worried about a doping violation

〇 Not effective

〇 Didn’t need it

〇 Experienced negative side effects

〇 Was told to discontinue use

〇 I have never discontinued the use of CBD

〇 Other (please specify) _____________

**20. If you do/did use Cannabidiol (CBD), did you inform a member of your integrated support team, sport support team or health care team? ***

***[Skip logic:*** *See question 16****]***

Please choose **only one** of the following:

〇 Yes

〇 No

〇 I do not have an integrated support team

**21. Did you document your Cannabidiol (CBD) use? ***

***[Skip logic:*** *See question 16****]***

Please choose **only one** of the following:

〇 Yes

〇 No

**22. How do/did you obtain Cannabidiol (CBD)?**

***[Skip logic:*** *See question 16****]***

Please choose **all** that apply:

〇 From a friend / family member

〇 From a teammate

〇 From a Health Canada approved retailer

〇 From a heath care facility/prescription (e.g., pharmacy, clinic)

〇 Online through a provincial retailer

〇 Online through another source

〇 Other (please specify) _________________

**23. When during your competition cycle do/did you take Cannabidiol (CBD)? ***

***[Skip logic:*** *See question 16****]***

Please choose **only one** of the following:

〇 In season, outside of competition

〇 In season, during competition

〇 Off season

〇 Other (please specify) ____________

**24. In relation to your training, when do/did you typically consume Cannabidiol (CBD)? ***

***[Skip logic:*** *See question 16****]***

Please choose **all** that apply:

〇 Before training

〇 During training

〇 After training

〇 In the evening prior to going to bed

〇 Other (please specify) ______________

**25. If you do/did use Cannabidiol (CBD) during competition, how do you feel it affected your performance? It made my performance… ***

***[Skip logic:*** *See question 16****]***

Please choose **only one** of the following:

〇 Much better

〇 Slightly better

〇 About the same

〇 Slightly worse

〇 Much worse

**26. In the following questions we are interested in your REASONS for taking Cannabidiol (CBD). For each statement, you take/took Cannabidiol (CBD) to: ***

***[Skip logic:*** *See question 16****]***

Please choose the appropriate response for each item:

|  | Strongly Disagree | Disagree | Agree | Strongly Agree |
| --- | --- | --- | --- | --- |
| To improve my sleep | 〇 | 〇 | 〇 | 〇 |
| To enhance physical recovery from training/competition | 〇 | 〇 | 〇 | 〇 |
| To enhance mental recovery from training/competition | 〇 | 〇 | 〇 | 〇 |
| To reduce acute pain from competition | 〇 | 〇 | 〇 | 〇 |
| To become more competitive (or to remain competitive) in my sport | 〇 | 〇 | 〇 | 〇 |
| To reduce pain from training | 〇 | 〇 | 〇 | 〇 |
| To reduce anxiety | 〇 | 〇 | 〇 | 〇 |
| To reduce feelings of depression | 〇 | 〇 | 〇 | 〇 |
| To improve physical performance | 〇 | 〇 | 〇 | 〇 |
| To improve focus/mental performance | 〇 | 〇 | 〇 | 〇 |
| To improve relaxation | 〇 | 〇 | 〇 | 〇 |

**27. If any, what other potential benefit(s) beyond those listed in question 26, have you experienced while using Cannabidiol (CBD)?**

***[Skip logic:*** *See question 16****]***

Please write your answer here:

|  |
| --- |

**28. If you have taken Cannabidiol (CBD), please indicate your level of agreement with the following statements: ***

***[Skip logic:*** *See question 16****]***

Please choose the appropriate response for each item:

|  | Strongly Disagree | Disagree | Agree | Strongly Agree |
| --- | --- | --- | --- | --- |
| I have/had benefits from taking Cannabidiol (CBD) | 〇 | 〇 | 〇 | 〇 |
| I think Cannabidiol (CBD) is relatively safe to use | 〇 | 〇 | 〇 | 〇 |
| I will likely continue to use Cannabidiol (CBD) in the future | 〇 | 〇 | 〇 | 〇 |
| I’m looking at Cannabidiol (CBD) as an alternative to pain medications (e.g., Ibuprofen, Tylenol)  due to concerns over their side effect | 〇 | 〇 | 〇 | 〇 |
| I am worried about anti-doping violations with cannabis products like Cannabidiol (CBD) | 〇 | 〇 | 〇 | 〇 |
| Although not a banned substance, I am reluctant to disclose my Cannabidiol (CBD) use | 〇 | 〇 | 〇 | 〇 |

**29. At what age did you start taking Cannabidiol (CBD)? ***

***[Skip logic:*** *See question 16****]***

Please write your answer here:

|  |
| --- |

**30. How many times per month do you/did you use Cannabidiol (CBD)? ***

***[Skip logic:*** *See question 16****]***

Please choose **only one** of the following:

〇 Less than once, on average

〇 1

〇 2

〇 3

〇 4

〇 5

〇 6

〇 7

〇 8

〇 9

〇 10

〇 11

〇 12

〇 13

〇 14

〇 15

〇 16

〇 17

〇 18

〇 19

〇 20

〇 21

〇 22

〇 23

〇 24

〇 25

〇 26

〇 27

〇 28

〇 29

〇 30

〇 Over 30 times

〇 Other (please specify) ______________

**31. How many times a day do/did you take Cannabidiol (CBD) at most? ***

***[Skip logic:*** *See question 16****]***

Please choose **only one** of the following:

〇 Once daily

〇 Twice daily

〇 Three times daily

〇 Four times or more each day

**32.** **How frequently do you take Cannabidiol (CBD)?**

***[Skip logic:*** *See question 16****]***

Please choose **only one** of the following:
〇 A few days at a time

〇 A few weeks at a time

〇 A few months at a time

〇 Almost always

〇 On and off / inconsistently

〇 Other (please specify) _______________

**33. What brand(s) of Cannabidiol (CBD) have you used? ***

**(If multiple, separate by a comma)**

***[Skip logic:*** *See question 16****]***

Please write your answer here:

|  |
| --- |

**34. To your knowledge, are all the brand(s) of Cannabidiol (CBD) product(s) you use "third-party" batch-tested (e.g., by NSF, Informed Sport, or Informed Choice) for contamination with other prohibited substances? ***

***[Skip logic:*** *See question 16****]***

Please choose **only one** of the following:

〇 Yes

〇 No

〇 Some are, and other aren’t

〇 I do not know

〇 Other (please specify) ____________

**35. How do/did you use Cannabidiol (CBD)? ***

***[Skip logic:*** *See question 16****]***

Please choose **all** that apply:

〇 Inhalation (e.g., dry herb, vaporizer, concentrates)

〇 Tincture/Oil

〇 Softgels/Capsules

〇 Confectionary/Edible

〇 Specific Sport Supplement with CBD

〇 Beverage

〇 Topical (e.g., cream)

〇 Other (please specify) ____________

**36. How much do/did you typically spend on Cannabidiol (CBD) products every month (all amounts are in Canadian dollar)? ***

***[Skip logic:*** *See question 16****]***

Please choose **only one** of the following:

〇 Less than $10

〇 $10 - $20

〇 $20 - $50

〇 $ 50 - $100

〇 Over $100

〇 Not sure

**37. What dose of Cannabidiol (CBD) did/do you take at a time? ***

***[Skip logic:*** *See question 16****]***

Please choose **only one** of the following:

〇 Less than 5 mg

〇 5-10 mg

〇 10-20 mg

〇 20-50 mg

〇 50-100 mg

〇 Over 100 mg

〇 Not sure

〇 Other (please specify) ____________

**38. How sure are you that this is the optimal dose of Cannabidiol (CBD) for you? ***

***[Skip logic:*** *See question 16****]***

Please choose **only one** of the following:

| 0% | 10% | 20% | 30% | 40% | 50% | 60% | 70% | 80% | 90% | 100% |
| --- | --- | --- | --- | --- | --- | --- | --- | --- | --- | --- |
| 〇 | 〇 | 〇 | 〇 | 〇 | 〇 | 〇 | 〇 | 〇 | 〇 | 〇 |

**39. How did you determine the dose of Cannabidiol (CBD) you took/take? ***

***[Skip logic:*** *See question 16****]***

Please choose **all** that apply:

〇 Trial and error

〇 Internet

〇 Package labelling

〇 Professional guidelines through a practitioner

〇 Never found an effective dose

〇 Other (please specify) ____________

**40. How often did you/do you experience adverse effect(s) from your use of Cannabidiol (CBD)? ***

**(Based on your answer choice, certain subsequent questions may or may not be presented)**

***[Skip logic:*** *See question 16****]***

***[Skip logic:*** *Do not* *show questions 41 and 42 if the athlete answers “Never” on question 40****]***

Please choose **only one** of the following:

| Never | Rarely | Sometimes | Often | Always |
| --- | --- | --- | --- | --- |
| 〇 | 〇 | 〇 | 〇 | 〇 |

**41. What side effect(s) did you experience? ***

***[Skip logic:*** *See question 16****]***

***[Skip logic:*** *See question 40****]***

Please choose **all** that apply:

〇 Dizziness

〇 Dry mouth

〇 Diarrhea

〇 Reduced appetite

〇 Drowsiness/fatigue

〇 Gastrointestinal side effects

〇 Other (please specify) ________________

**42. Please read each statement and decide the extent to which you agree or disagree: ***

***[Skip logic:*** *See question 16****]***

***[Skip logic:*** *See question 40****]***

Please choose the appropriate response for each item:

|  | Strongly Disagree | Disagree | Agree | Strongly Agree |
| --- | --- | --- | --- | --- |
| I had significant side effects from taking Cannabidiol (CBD) | 〇 | 〇 | 〇 | 〇 |
| The side effects from taking Cannabidiol (CBD) were bothersome | 〇 | 〇 | 〇 | 〇 |
| The positive effects of taking Cannabidiol (CBD) outweigh the negative effects | 〇 | 〇 | 〇 | 〇 |
| I do not intend to take Cannabidiol (CBD) again due to the side effects | 〇 | 〇 | 〇 | 〇 |

**Section D. Assumptions about CBD use**

**43. Which of these would be reasons for you not to take Cannabidiol (CBD)?**Please choose **all** that apply:

〇 Do not see a need for it

〇 Do not know enough about it

〇 Concerned about anti-doping violation

〇 Not aware of the possible benefits

〇 Was advised against it

〇 Expensive

〇 I don’t see any reason not to take CBD

〇 Other (please specify) ______________

**44. How likely is it that you will start, restart, or continue to use use Cannabidiol (CBD) within the next 12 months? ***

Please choose **only one** of the following:

| Very unlikely | Unlikely | Likely | Very likely |
| --- | --- | --- | --- |
| 〇 | 〇 | 〇 | 〇 |

**45. How many of your teammates/people in your training group do you think use Cannabidiol (CBD)? ***

Please choose **only one** of the following:

| None | Very few | Some | Many | Most |
| --- | --- | --- | --- | --- |
| 〇 | 〇 | 〇 | 〇 | 〇 |

**46. How many of your competitors do you think use Cannabidiol (CBD)? ***

Please choose **only one** of the following:

| None | Very few | Some | Many | Most |
| --- | --- | --- | --- | --- |
| 〇 | 〇 | 〇 | 〇 | 〇 |

**47. How many people at the highest level of your sport (i.e. people who compete internationally) do you think use Cannabidiol (CBD)? ***

Please choose **only one** of the following:

| None | Very few | Some | Many | Most |
| --- | --- | --- | --- | --- |
| 〇 | 〇 | 〇 | 〇 | 〇 |

**48. For each of the following, indicate how often Cannabidiol (CBD) is
discussed ***

Please choose the appropriate response for each item:

|  | Never | Rarely | Sometimes | Often | Always |
| --- | --- | --- | --- | --- | --- |
| How often do you talk about Cannabidiol (CBD) with your teammates? | 〇 | 〇 | 〇 | 〇 | 〇 |
| How often do you talk about Cannabidiol (CBD) with your coach? | 〇 | 〇 | 〇 | 〇 | 〇 |
| How often do you talk about Cannabidiol (CBD) with members of your integrated support team? | 〇 | 〇 | 〇 | 〇 | 〇 |

**49. Consider each of the following scenarios. How likely is it that you would start, continue, or increase the use of Cannabidiol (CBD) if… ***

Please choose the appropriate response for each item:

|  | Very unlikely | Unlikely | Likely | Very likely |
| --- | --- | --- | --- | --- |
| It was prescribed/encouraged by my coach | 〇 | 〇 | 〇 | 〇 |
| It was prescribed/encouraged by my nutritionist | 〇 | 〇 | 〇 | 〇 |
| It was prescribed/encouraged by my sports medicine doctor | 〇 | 〇 | 〇 | 〇 |
| It had been tested and confirmed to be free from banned substances (e.g., by NSF, Informed Sport, or Informed Choice) | 〇 | 〇 | 〇 | 〇 |
| It was confirmed by researchers to be safe | 〇 | 〇 | 〇 | 〇 |
| It was more affordable | 〇 | 〇 | 〇 | 〇 |
| It was more accessible | 〇 | 〇 | 〇 | 〇 |
| It was more acceptable by the sporting community | 〇 | 〇 | 〇 | 〇 |
| It was encouraged by a CBD company sponsor | 〇 | 〇 | 〇 | 〇 |
| The side effects were milder / more acceptable | 〇 | 〇 | 〇 | 〇 |

**50. What other scenarios would make you more likely to start using Cannabidiol (CBD)?**Please write your answer here:

|  |
| --- |

**51. The following are scenarios regarding Cannabidiol (CBD) research. Which scenarios do you think would lead to more widespread acceptance among athletes? ***

Please choose the appropriate response for each item:

|  | Very unlikely | Unlikely | Likely | Very likely |
| --- | --- | --- | --- | --- |
| It was confirmed by researchers to be effective at improving recovery | 〇 | 〇 | 〇 | 〇 |
| It was confirmed by researchers to be effective at improving sleep | 〇 | 〇 | 〇 | 〇 |
| It was confirmed by researchers to be effective at improving exercise performance | 〇 | 〇 | 〇 | 〇 |
| It was confirmed by researchers to be effective at decreasing anxiety | 〇 | 〇 | 〇 | 〇 |

**Section E. Comments**

**52. Please include below any further comments in regard to Cannabidiol (CBD) that you may have:**

Please write your answer here:

|  |
| --- |
