# Supplementary material for: Cannabidiol use among elite-level Canadian athletes: the pursuit of improved sleep, pain relief, and enhanced recovery
Source: Front Nutr. 2025 Dec 5;12:1711773. doi: 10.3389/fnut.2025.1711773 (PMC12715612; doi:10.3389/fnut.2025.1711773)
Supplement: Supplementary file 2 [file Table_2.docx]

**Questionnaire de McGill sur le Cannabidiol (CBD)**

**Étude de l'utilisation du Cannabidiol (CBD) par les athlètes d’élites au Canada**

Sondage de 52 questions

**Section A. Informations sur l’athlète**

**1**. **Quel est votre âge? ***

Veuillez écrire votre réponse ici :

|  |
| --- |

**2. Quel sexe vous a été attribué à la naissance? ***

**(Ex. femme, homme, préfère ne pas dire, etc.)**
Veuillez écrire votre réponse ici :

|  |
| --- |

**3. Quel est votre identité de genre actuelle?** *

**(Ex. femme, homme, autre)**

Veuillez écrire votre réponse ici:

|  |
| --- |

**4. À quel niveau faites-vous de la compétition? ***

Veuillez choisir **toutes** les réponses qui s’appliquent:

〇 Provincial

〇 National
〇 Niveau international/olympique

**5. Participez-vous à des compétitions de USport? ***

Veuillez sélectionner **une seule** des propositions suivantes :

〇 Oui

〇 Non

〇 Ne sais pas

**6. Recevez-vous un soutien en sciences du sport et/ou un soutien médical par l'intermédiaire de l’Institut Canadienne du Sport, de l'Institut National du Sport du Québec ou d'un Centre Canadien du Sport? ***
Veuillez sélectionner **une seule** des propositions suivantes :

〇 Oui

〇 Non

**7. Comment recevez-vous du financement pour votre sport? ***

Veuillez choisir **toutes** les réponses qui s’appliquent:

〇 Je me finance moi-même

〇 Je suis un athlète parrainé par le gouvernement

〇 Je suis un athlète individuel parrainé par une entreprise

〇 Je fais partie d'une équipe sponsorisée par une entreprise

〇 Je suis un athlète professionnel

**8. Êtes-vous un para athlète? ***

Veuillez sélectionner **une seule** des propositions suivantes :

〇 Oui

〇 Non

**9. Quel est le principal sport que vous pratiquez en compétition? ***

Veuillez écrire votre réponse ici:

|  |
| --- |

**10. Pratiquez-vous d'autres sports en compétition? ***

**(Selon votre choix de réponse, certaines questions subséquentes peuvent apparaître ou non)**

***[Skip logic:*** *Montrer la question 11 si l’athlète répond « Oui » à la question 10****]***

Veuillez sélectionner **une seule** des propositions suivantes :
〇 Oui

〇 Non

**11. Quels autres sports pratiquez-vous en compétition?**

**(S'il y a plusieurs sports, séparer chacun des sports avec des virgules)
*[Skip logic:*** *Voir question 10****]***

Veuillez écrire votre réponse ici:

|  |
| --- |

**12. Avec combien de personnes vous entraînez-vous habituellement? ***

Veuillez sélectionner **une seule** des propositions suivantes :

〇 Zéro (je m'entraîne seul)

〇 1

〇 2

〇 3

〇 4

〇 5

〇 6

〇 7

〇 8

〇 9

〇 10 et plus

**Section B. Connaissances sur le CBD**

**13. Aviez-vous déjà entendu parler du Cannabidiol (CBD) avant de remplir ce questionnaire? ***

**(Selon votre choix de réponse, certaines questions subséquentes peuvent apparaître ou non)**

***[Skip logic:*** *Montrer la question 14 si l’athlète répond « Oui » à la question 13****]***

Veuillez sélectionner **une seule** des propositions suivantes :

〇 Oui

〇 Non

**14. Comment avez-vous entendu parler du Cannabidiol (CBD)?** *

**(Selon votre choix de réponse, certaines questions subséquentes peuvent apparaître ou non)**

***[Skip logic:*** *Voir question 13****]***

***[Skip logic:*** *Montrer la question 15 si l’athlète répond « Internet » à la question 14****]***

Veuillez choisir **toutes** les réponses qui s’appliquent:

〇 Internet (i.e., les médias sociaux, les médias d'information, ou la publicité)

〇 Nutritionniste / Diététicien(ne)

〇 Médecin

〇 Autre praticien (Ex. massothérapeute, physiothérapeute, chiropraticien)

〇 Ami(e)

〇 Coéquipier / Coéquipière

〇 Autre athlète

〇 Membre de la famille

〇 Entraineur

〇 Autre (veuillez préciser) _________________

**15. Si vous avez entendu parler du Cannabidiol (CBD) sur internet, quelle source avez-vous consulté?**

***[Skip logic:*** *Voir question 14****]***

Veuillez choisir **toutes** les réponses qui s’appliquent:
〇 Nouvelles (Ex. cbc.ca)

〇 Blogue

〇 Forum (Ex. Reddit)

〇 Médias sociaux (Ex. Facebook, Instagram)

〇 Annonce publicitaire sur Internet

**Section C. Consommation du CBD**

**16. Avez-vous déjà utilisé du Cannabidiol (CBD)?**

**(Selon votre choix de réponse, certaines questions subséquentes peuvent apparaître ou non)**

***[Skip logic:*** *Montrer les questions 17 à 42 si l’athlète répond « Oui » à la question 16****]***

Veuillez sélectionner **une seule** des propositions suivantes :

〇 Oui

〇 Non

**17. Utilisez-vous actuellement du Cannabidiol (CBD)? ***

***[Skip logic:*** *Voir question 16****]***

Veuillez sélectionner **une seule** des propositions suivantes :

〇 Oui

〇 Non

**18.** **Depuis combien de temps utilisez-vous du Cannabidiol (CBD)? ***

**(ex. 1 an et 6 mois)**
***[Skip logic:*** *Voir question 16****]***

Veuillez écrire votre réponse ici:

|  |
| --- |

**19. Si vous étiez utilisateur du Cannabidiol (CBD) dans le passé et avez soit arrêté l’usage complètement ou arrêté pour une période de temps, quelles sont les raisons de votre arrêt? ***

***[Skip logic:*** *Voir question 16****]***Veuillez choisir **toutes** les réponses qui s’appliquent:
〇 Trop dispendieux

〇 Inquiétudes concernant la violation des règles antidopage

〇 Pas efficace

〇 Pas besoin

〇 Subi des effets secondaires négatifs

〇 On m’a recommandé de discontinuer l’usage

〇 Je n'ai jamais cesser l'usage du CBD

〇 Autre (veuillez préciser) _____________

**20. Si vous utilisez/avez utilisé du Cannabidiol (CBD), avez-vous informé un membre de votre équipe de soutien intégré, de votre équipe de soutien sportif ou de votre équipe de soins de santé? ***

***[Skip logic:*** *Voir question 16****]***

Veuillez sélectionner **une seule** des propositions suivantes :

**〇** Oui **〇** Non **〇** Je n’ai pas d'équipe de soutien intégré

**21. Avez-vous documenté votre consommation de Cannabidiol (CBD)? ***

***[Skip logic:*** *Voir question 16****]***

Veuillez sélectionner **une seule** des propositions suivantes :

**〇** Oui **〇** Non

**22. Comment obtenez-vous ou avez-vous obtenu du Cannabidiol (CBD)? ***

***[Skip logic:*** *Voir question 16****]***Veuillez choisir **toutes** les réponses qui s’appliquent:

**〇** D’un ami / membre de la famille **〇** D’un(e) coéquipier / coéquipière
**〇** D’un détaillant approuvé par Santé Canada
**〇** D'un établissement de soins de santé ou d'une ordonnance (Ex. pharmacie, clinique)
**〇** En ligne chez un détaillant provincial
**〇** En ligne par une autre source
**〇** Autre (veuillez préciser) _________________

**23. Durant vos cycles de compétition, quand prenez-vous ou avez-vous pris du Cannabidiol (CBD)? ***

***[Skip logic:*** *Voir question 16****]***Veuillez choisir **toutes** les réponses qui s’appliquent:

〇 En saison, hors compétition
〇 En saison, pendant la compétition
〇 Hors saison
〇 Autre (veuillez préciser) _________________

**24. Dans le cadre de votre entraînement, quand consommez-vous ou avez-vous consommé du Cannabidiol (CBD)? ***

***[Skip logic:*** *Voir question 16****]***

Veuillez choisir **toutes** les réponses qui s’appliquent:

〇 Avant l'entraînement
〇 Pendant l'entraînement
〇 Après l'entraînement
〇 Le soir avant d'aller au lit
〇 Autre (veuillez préciser) _________________

**25. Si vous utilisez ou avez utilisé du Cannabidiol (CBD) pendant la compétition, comment pensez-vous que cela a affecté votre performance ? Ma performance a été… ***

***[Skip logic:*** *Voir question 16****]***

Veuillez sélectionner **une seule** des propositions suivantes :

〇 Beaucoup meilleure

〇 Un peu meilleure

〇 Semblablement la même

〇 Plus pire

〇 Beaucoup plus pire

**26. Dans les questions suivantes, nous nous intéressons aux RAISONS pour lesquelles vous prenez du Cannabidiol (CBD). Pour chaque affirmation, vous prenez/avez pris du Cannabidiol (CBD):**

***[Skip logic:*** *Voir question 16****]***

Choisissez la réponse appropriée pour chaque élément :

|  | Fortement en désaccord |  | En désaccord | En accord | Fortement en accord |
| --- | --- | --- | --- | --- | --- |
| Pour améliorer mon sommeil | 〇 |  | 〇 | 〇 | 〇 |
| Pour améliorer la récupération physique après un entraînement ou une compétition | 〇 |  | 〇 | 〇 | 〇 |
| Pour améliorer la récupération mentale après un entraînement/une compétition | 〇 |  | 〇 | 〇 | 〇 |
| Pour réduire la douleur aiguë de la compétition | 〇 |  | 〇 | 〇 | 〇 |
| Pour devenir plus compétitif (ou rester compétitif) dans mon sport | 〇 |  | 〇 | 〇 | 〇 |
| Pour réduire les douleurs d’entrainement | 〇 |  | 〇 | 〇 | 〇 |
| Pour réduire l'anxiété | 〇 |  | 〇 | 〇 | 〇 |
| Pour réduire les sensations de dépression | 〇 |  | 〇 | 〇 | 〇 |
| Pour améliorer la performance physique | 〇 |  | 〇 | 〇 | 〇 |
| Pour améliorer ma concentration/performance mentale | 〇 |  | 〇 | 〇 | 〇 |
| Pour améliorer la relaxation | 〇 |  | 〇 | 〇 | 〇 |

**27. Si présent, quel(s) autre(s) bénéfice(s), en dehors de ceux énumérés dans la question 26, avez-vous constaté en utilisant le Cannabidiol (CBD)?**

***[Skip logic:*** *Voir question 16****]***

Veuillez écrire votre réponse ici:

|  |
| --- |

**28. Si vous avez pris du Cannabidiol (CBD), veuillez indiquer votre degré d'accord avec les déclarations suivantes :**

***[Skip logic:*** *Voir question 16****]***

Choisissez la réponse appropriée pour chaque élément :

|  | Fortement en désaccord | En désaccord | En accord | Fortement en accord |
| --- | --- | --- | --- | --- |
| J’ai / j’avais des bénéfices en prenant du Cannabidiol (CBD) | 〇 | 〇 | 〇 | 〇 |
| Je pense que l’usage du Cannabidiol (CBD) est relativement sûr, autrement dit sans danger} | 〇 | 〇 | 〇 | 〇 |
| Je vais probablement continuer à utiliser du Cannabidiol (CBD) à l'avenir | 〇 | 〇 | 〇 | 〇 |
| Je considère le Cannabidiol (CBD) comme une alternative aux médicaments contre la douleur (e.g., Ibuprofen, Tylenol) due à de l’inquiétude concernant leurs effets secondaires | 〇 | 〇 | 〇 | 〇 |
| Je m'inquiète des violations des règles antidopage avec les produits à base de cannabis comme le Cannabidiol (CBD) | 〇 | 〇 | 〇 | 〇 |
| Bien qu'il ne s'agisse pas d'une substance interdite, je suis réticent à divulguer ma consommation de Cannabidiol (CBD) | 〇 | 〇 | 〇 | 〇 |

**29. À quel âge avez-vous commencé à prendre du Cannabidiol (CBD)? ***

***[Skip logic:*** *Voir question 16****]***

Veuillez écrire votre réponse ici:

|  |
| --- |

**30. Combien de fois par mois utilisez-vous/avez-vous utilisé du Cannabidiol (CBD)? ***

***[Skip logic:*** *Voir question 16****]***Veuillez sélectionner **une seule** des propositions suivantes :

〇 Moins qu’une fois, en moyenne

〇 1

〇 2

〇 3

〇 4

〇 5

〇 6

〇 7

〇 8

〇 9

〇 10

〇 11

〇 12

〇 13

〇 14

〇 15

〇 16

〇 17

〇 18

〇 19

〇 20

〇 21

〇 22

〇 23

〇 24

〇 25

〇 26

〇 27

〇 28

〇 29

〇 30

〇 Plus que 30 fois

〇 Autre (veuillez préciser) _________________

**31. Combien de fois par jour prenez-vous/avez-vous pris du Cannabidiol (CBD) au plus? ***

***[Skip logic:*** *Voir question 16****]***

Veuillez sélectionner **une seule** des propositions suivantes :

**〇** Une fois par jour
〇 Deux fois par jour
〇 Trois fois par jour
**〇** Quatre fois ou plus, par jour

**32. À quelle fréquence consommez-vous du Cannabidiol (CBD)? ***

***[Skip logic:*** *Voir question 16****]***

Veuillez sélectionner **une seule** des propositions suivantes :

〇 Quelques jours à la fois
〇 Quelques semaines à la fois
〇 Quelques mois à la fois
〇 Presque toujours
〇 Par intermittence / de façon irrégulière
〇 Autre (veuillez préciser) _______________

**33. Quelle(s) marque(s) de Cannabidiol (CBD) avez-vous utilisée? ***

**(Si plusieurs, séparer avec des virgules)**

***[Skip logic:*** *Voir question 16****]***

Veuillez écrire votre réponse ici:

|  |
| --- |

**34. Selon vous, toutes les marque(s) de produit(s) de Cannabidiol (CBD) que vous utilisez ont-elles fait l'objet d'un contrôle de lot par une tierce partie (Ex. par NSF, Informed Sport, ou Informed Choice) pour détecter toute contamination par d'autres substances interdites? ***

***[Skip logic:*** *Voir question 16****]***

Veuillez sélectionner **une seule** des propositions suivantes :

**〇** Oui **〇** Non

**〇** Certains oui, et d’autres non **〇** Je ne sais pas **〇** Autre (veuillez préciser) ____________

**35. Comment utilisez-vous/avez-vous utilisé le Cannabidiol (CBD)? ***

***[Skip logic:*** *Voir question 16****]***
Veuillez choisir **toutes** les réponses qui s’appliquent:

**〇** Inhalation (Ex. herbe sèche, vaporisateur, concentrés)
〇 Teinture/Huile
〇 Gélules/Capsules
〇 Confiserie/Comestible « Edible »
〇 Supplément sportif spécifique avec CBD
〇 Boisson
〇 Topique (ex. crème)
〇 Autre (veuillez préciser) ____________

**36. Combien dépensez-vous habituellement /avez-vous dépensé en produits à base de Cannabidiol (CBD) chaque mois (tous les montants sont en dollar Canadien)? ***

***[Skip logic:*** *Voir question 16****]***

Veuillez sélectionner **une seule** des propositions suivantes :

〇 Moins que 10$
〇 10$-20$
〇 20$-50$
〇 50$-100$
〇 Plus que 100$
〇 Pas certain(e)

**37. Quelle dose de Cannabidiol (CBD) avez-vous pris/prenez-vous à la fois? ***

***[Skip logic:*** *Voir question 16****]***

Veuillez sélectionner **une seule** des propositions suivantes :

〇 Moins que 5 mg
〇 5-10 mg
〇 10-20 mg
〇 20-50 mg
〇 50-100 mg
〇 Plus que 100 mg
〇 Pas certain(e)

〇 Autre (veuillez préciser) ____________

**38. Combien êtes-vous certain que c'est la dose optimale de Cannabidiol (CBD) pour vous? ***

***[Skip logic:*** *Voir question 16****]***

Veuillez sélectionner **une seule** des propositions suivantes :

| 0% | 10% | 20% | 30% | 40% | 50% | 60% | 70% | 80% | 90% | 100% |
| --- | --- | --- | --- | --- | --- | --- | --- | --- | --- | --- |
| 〇 | 〇 | 〇 | 〇 | 〇 | 〇 | 〇 | 〇 | 〇 | 〇 | 〇 |

**39. Comment avez-vous déterminé la dose de Cannabidiol (CBD) que vous avez prise/pris? ***

***[Skip logic:*** *Voir question 16****]***

Veuillez choisir **toutes** les réponses qui s’appliquent:

〇 Essai et erreur
〇 Internet
〇 Étiquetage de l'emballage
〇 Directives professionnelles par l'intermédiaire d'un praticien
〇 Jamais trouvé de dose efficace
〇 Autre (veuillez préciser) ____________

**40. À quelle fréquence avez-vous ressenti ou ressentez-vous un ou des effets secondaires liés à votre consommation de Cannabidiol (CBD)? ***

**(Selon votre choix de réponse, certaines questions subséquentes peuvent apparaître ou non)**

***[Skip logic:*** *Voir question 16****]***

***[Skip logic:*** *Ne pas* *montrer les question 41 et 42 si l’athlète répond « Jamais » à la question 40****]***

Veuillez sélectionner **une seule** des propositions suivantes :

| Jamais | Rarement | Occasionnellement | Souvent | Toujours |
| --- | --- | --- | --- | --- |
| 〇 | 〇 | 〇 | 〇 | 〇 |

**41. Quel(s) effet(s) secondaire(s) avez-vous ressenti(s)? ***

***[Skip logic:*** *Voir question 16****]***

***[Skip logic:*** *Voir question 40****]***Veuillez choisir **toutes** les réponses qui s’appliquent:

〇 Vertiges
〇 Sécheresse de la bouche
〇 Diarrhée
〇 Diminution de l'appétit
〇 Somnolence/fatigue
〇 Effets secondaires gastro-intestinaux
〇 Autre (veuillez préciser) ________________

**42. Veuillez lire chaque affirmation et décider dans quelle mesure vous êtes d'accord ou non : ***

***[Skip logic:*** *Voir question 16****]***

***[Skip logic:*** *Voir question 40****]***

Choisissez la réponse appropriée pour chaque élément :

|  | Fortement en désaccord | En désaccord | En accord | Fortement en accord |
| --- | --- | --- | --- | --- |
| J'ai eu des effets secondaires importants en prenant du Cannabidiol (CBD) | 〇 | 〇 | 〇 | 〇 |
| Les effets secondaires de la prise de Cannabidiol (CBD) étaient dérangeant | 〇 | 〇 | 〇 | 〇 |
| Les effets positifs de la prise de Cannabidiol (CBD) l'emportent sur les effets négatifs | 〇 | 〇 | 〇 | 〇 |
| Je n'ai pas l'intention de reprendre du Cannabidiol (CBD) à cause des effets secondaires | 〇 | 〇 | 〇 | 〇 |

**Section D. Hypothèses sur l’utilisation du CBD**

**43. Lesquelles de ces raisons vous inciteraient à ne pas prendre ou à cesser l’usage du Cannabidiol (CBD)? ***

Veuillez choisir **toutes** les réponses qui s’appliquent:

〇 Je n'en vois pas la nécessité ou le besoin
〇 Je n'en sais pas assez sur le sujet
〇 Je suis préoccupé par une violation des règles antidopage
〇 Pas conscient des bénéfices possibles
〇 On me l'a déconseillé
〇 Coûteux

〇 Je ne trouve pas de raison pour cesser l’usage du CBD
〇 Autre (veuillez préciser) ______________

**44. Quelle est la probabilité que vous commencez, recommencez, ou continuez à utiliser du Cannabidiol (CBD) au cours des 12 prochains mois? ***

Veuillez sélectionner **une seule** des propositions suivantes :

| Très improbable | Improbable | Probable | Très probable |
| --- | --- | --- | --- |
| 〇 | 〇 | 〇 | 〇 |

**45. Selon vous, combien de vos coéquipiers ou de personnes de votre groupe d'entraînement utilisent du Cannabidiol (CBD)? ***

Veuillez sélectionner **une seule** des propositions suivantes :

| Aucun | Presque aucun | Quelques-uns | Plusieurs | La plupart |
| --- | --- | --- | --- | --- |
| 〇 | 〇 | 〇 | 〇 | 〇 |

**46. Selon vous, combien de vos compétiteurs utilisent du Cannabidiol (CBD)? ***

Veuillez sélectionner **une seule** des propositions suivantes :

| Aucun | Presque aucun | Quelques-uns | Plusieurs | La plupart |
| --- | --- | --- | --- | --- |
| 〇 | 〇 | 〇 | 〇 | 〇 |

**47. Selon vous, combien d’athlètes de haut niveau dans votre sport (c'est-à-dire des personnes qui participent à des compétitions internationales) utilisent du Cannabidiol (CBD)? ***

Veuillez sélectionner **une seule** des propositions suivantes :

| Aucun | Presque aucun | Quelques-uns | Plusieurs | La plupart |
| --- | --- | --- | --- | --- |
| 〇 | 〇 | 〇 | 〇 | 〇 |

**48. Pour chacun des éléments suivants, indiquez à quelle fréquence le Cannabidiol (CBD) est discuté ***

Choisissez la réponse appropriée pour chaque élément :

|  | Jamais | Rarement | Parfois | Souvent | Toujours |
| --- | --- | --- | --- | --- | --- |
| À quelle fréquence parlez-vous du Cannabidiol (CBD) avec vos coéquipiers ? | 〇 | 〇 | 〇 | 〇 | 〇 |
| À quelle fréquence parlez-vous du Cannabidiol (CBD) avec votre entraineur/coach ? | 〇 | 〇 | 〇 | 〇 | 〇 |
| À quelle fréquence parlez-vous du Cannabidiol (CBD) avec les membres de votre équipe de soutien  intégré ? | 〇 | 〇 | 〇 | 〇 | 〇 |

**49. Considérez chacun des scénarios suivants. Indiquez la probabilité que vous débutez, continuez, ou augmentez votre consommation du Cannabidiol (CBD) si… ***

Choisissez la réponse appropriée pour chaque élément :

|  | Très improbable | Improbable | Probable | Très probable |
| --- | --- | --- | --- | --- |
| Il a été prescrit/encouragé par mon entraîneur | 〇 | 〇 | 〇 | 〇 |
| Il a été prescrit/encouragé par mon nutritionniste | 〇 | 〇 | 〇 | 〇 |
| Il a été prescrit/encouragé par mon médecin du sport | 〇 | 〇 | 〇 | 〇 |
| Il a été testé et confirmé qu'il ne contient pas de substances interdites (e.g., par NSF, Informed Sport, ou Informed Choice) | 〇 | 〇 | 〇 | 〇 |
| Les chercheurs ont confirmé son innocuité (sans danger) | 〇 | 〇 | 〇 | 〇 |
| C'était plus abordable ($) | 〇 | 〇 | 〇 | 〇 |
| C'était plus accessible | 〇 | 〇 | 〇 | 〇 |
| C'était plus accepte par la communauté sportive | 〇 | 〇 | 〇 | 〇 |
| Il a été encouragé par un sponsor de l'entreprise CBD | 〇 | 〇 | 〇 | 〇 |
| Les effets secondaires étaient plus légers / plus acceptables | 〇 | 〇 | 〇 | 〇 |

**50. Quels autres scénarios vous inciteraient à commencer la consommation du Cannabidiol (CBD)?**

Veuillez écrire votre réponse ici:

|  |
| --- |

**51. Les scénarios suivants concernent la recherche sur le Cannabidiol (CBD). Selon vous, quels scénarios conduiraient à une acceptation plus large parmi les athlètes? ***

Choisissez la réponse appropriée pour chaque élément :

|  | Très improbable | Improbable | Probable | Très probable |
| --- | --- | --- | --- | --- |
| Les chercheurs ont confirmé son efficacité pour améliorer la récupération | 〇 | 〇 | 〇 | 〇 |
| Les chercheurs ont confirmé son efficacité pour améliorer le sommeil | 〇 | 〇 | 〇 | 〇 |
| Les chercheurs ont confirmé son efficacité pour améliorer la performance physique | 〇 | 〇 | 〇 | 〇 |
| Les chercheurs ont confirmé son efficacité pour réduire l'anxiété | 〇 | 〇 | 〇 | 〇 |

**Section E. Commentaires**

**52. Veuillez inclure ci-dessous tout autre commentaire concernant le Cannabidiol (CBD) que vous pourriez avoir :**

Veuillez écrire votre réponse ici:

|  |
| --- |
