# Supplementary material for: Cannabidiol use among elite-level Canadian athletes: the pursuit of improved sleep, pain relief, and enhanced recovery
Source: Front Nutr. 2025 Dec 5;12:1711773. doi: 10.3389/fnut.2025.1711773 (PMC12715612; doi:10.3389/fnut.2025.1711773)

frontiers

Supplemental Material:

RESULTS

Supplemental Figure Legends

Supplemental Figure 1. Flowchart depicting participant progression through the survey study. CBD; cannabidiol.

Supplemental Figure 2. Proportions reported by CBD users (n = 30) for (a) the most common modes of CBD consumption, (b) methods used to determine an effective dose, and (c) certainty in achieving optimal CBD dosing. CBD; cannabidiol.

Supplemental Figure 3. Proportions of CBD users (n = 30) reporting different timing of CBD use in relation to (a) training and (b) competition cycles. CBD; cannabidiol.

Supplemental Figure 4. Orderly proportions of reasons for never using or discontinuing CBD use among most participants (n = 79). CBD; cannabidiol.

Supplemental Figure 1.

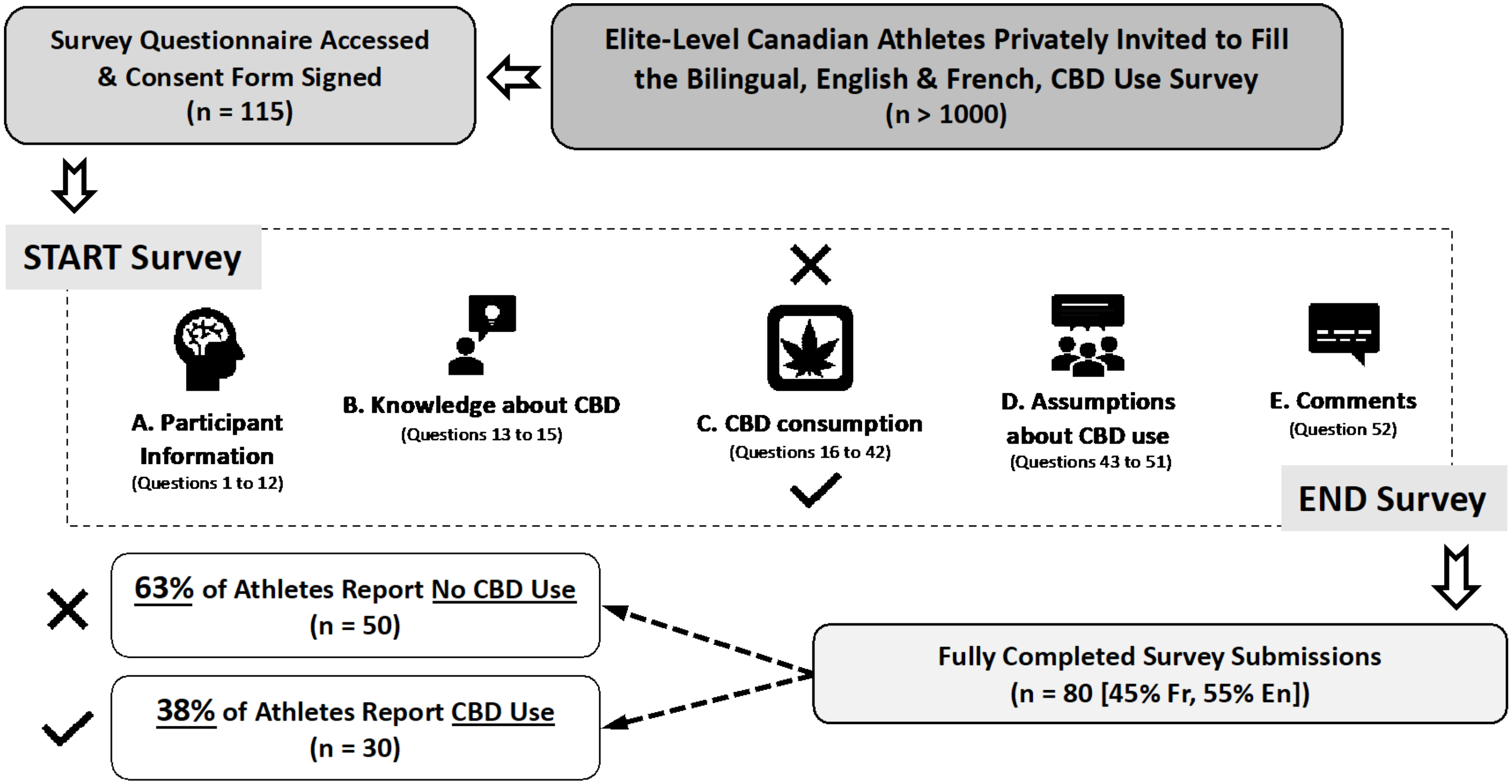

Supplemental Figure 2.

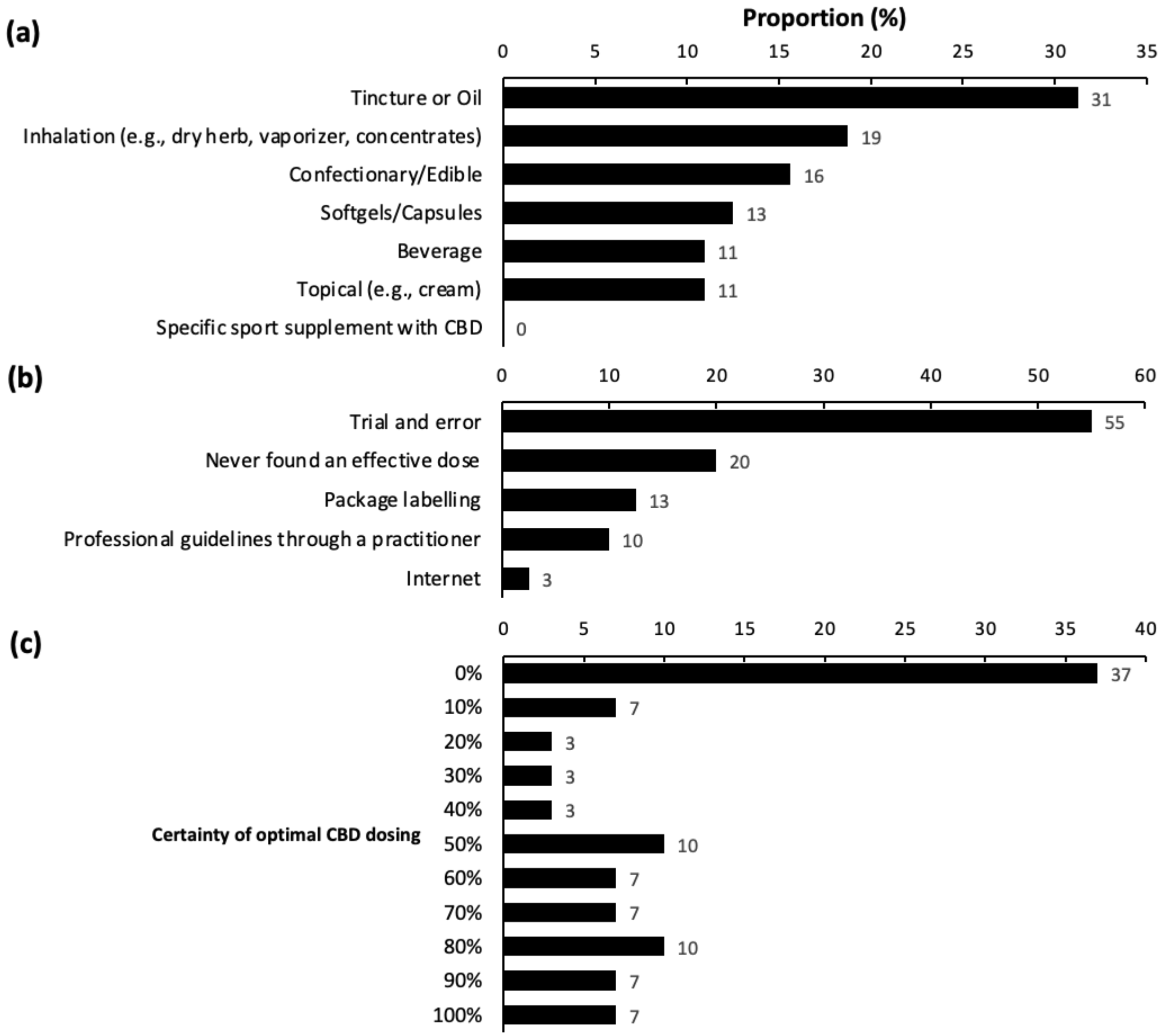

Supplemental Figure 3.

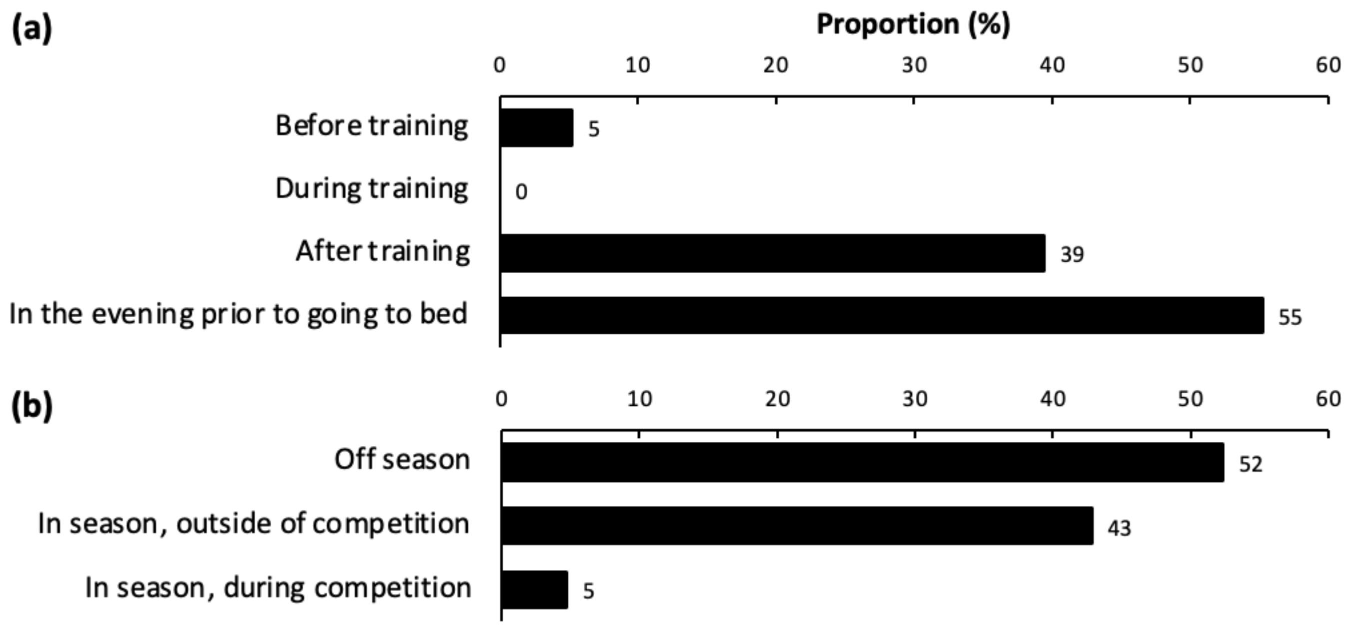

Supplemental Figure 4.

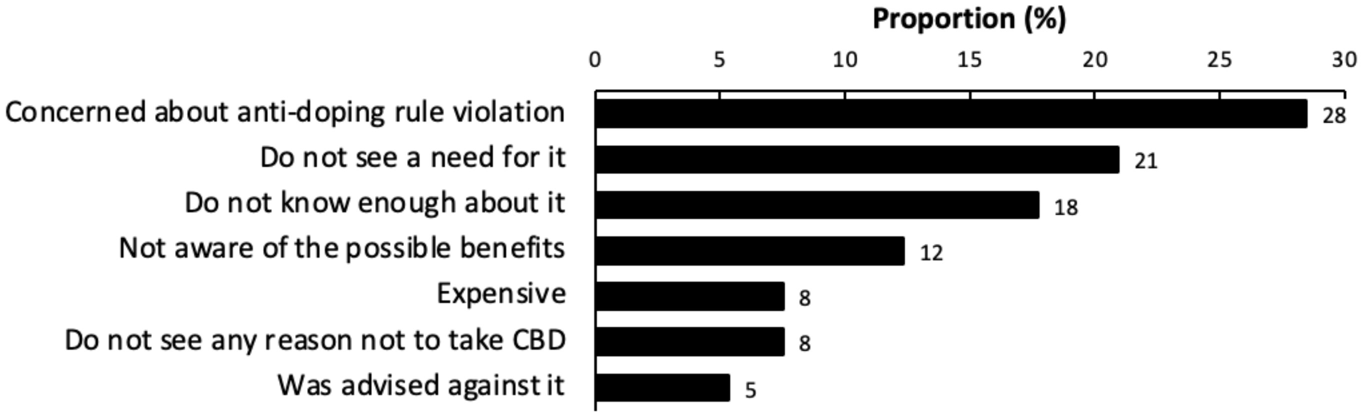

Supplement: Supplementary file 3 [file Image_1.pdf]
